# Did Children Interact With Their Personified Objects During the COVID-19 Pandemic?

**DOI:** 10.1177/02762366211034402

**Published:** 2022-03

**Authors:** Masanori Yamaguchi, Yusuke Moriguchi

**Affiliations:** 1 Department of Education, Kyoto University, Japan; 2 Japan Society for the Promotion of Science, Tokyo, Japan; 3 Department of Letters, Kyoto University, Japan

**Keywords:** personified objects, invisible friends, social isolation, pretend play

## Abstract

Although previous studies revealed the characteristics of children with imaginary companions, the characteristics of children alone could not explain why some children create and interact with imaginary companions. The current cross-sectional study examined the impact of the situational factors, decreased opportunities to meet and play with real playmate due to the COVID-19 pandemic, on the prevalence of imaginary companions. Five hundred sixty caregivers of children aged 2–9 years (half of them were girls) were asked whether their children currently had imaginary companions (personified objects and invisible friends) before (September 2019) and during the pandemic (April 2020). The logistic regression model showed that only the prevalence of personified objects increased during the pandemic, *OR* = 2.01, 95%CI [1.34, 3.00], even when potential variables were controlled. The results suggest that children more frequently played with their personified objects during the pandemic compared to before the pandemic.

Imaginary companions (ICs), “an imagined character that is talked about or interacted with on a regular basis” (Tahiroglu & Taylor, 2019, p. 2), might function as a playmate when there is no available real playmate (Taylor, 1999). Some children create ICs by attributing personality traits to their special stuffed animals or dolls (i.e., personified objects; POs), whereas others might do so to invisible entities (i.e., invisible friends; IFs) (Gleason et al., 2000; Singer & Singer, 1990; Taylor, 1999). Developmental researchers have tackled the question of why some children create and interact with ICs. Previous studies revealed the characteristics of children who have ICs. However, it seems that children’s characteristics alone did not determine the creation of ICs. In the current study, we explore a situational factor that stimulates creation of ICs.

Recent studies revealed the characteristics of children with ICs based on the literatures of typically developing children. For example, Gleason (2004) showed that the number of reciprocal friends did not differ between children with ICs and those without ICs. Moreover, a recent report showed that children with ICs received more positive and popularity nominations, fewer negative and unpopularity nominations, and were rated higher in social competence than those without ICs (Lin et al., 2018). Other studies also showed that children with ICs were outgoing (Roby & Kidd, 2008), liked to play with other children (Mauro, 1991), and were possibly more interested in social interactions than those without ICs (Tahiroglu & Taylor, 2019). Thus, these findings indicated that children with ICs can be characterized as being interested in social interaction, are socially competent, and surrounded by friends.

It is difficult to consider why these children need to create ICs based only on the children’s characteristics summarized above. We propose that creation of ICs is stimulated not only by children’s characteristics, but also by situational factors. Previous studies suggest that creation and interaction with ICs might be motivated by multiple situations: including when avoiding blame, overcoming fears, under stress, communicating with others (especially about sensitive topic for a child), coping with problems or trauma, and being bored and alone (Majors, 2013; Taylor, 1999). Given that ICs must be kept for a certain period (at least a month) by definition, the situations that frequently occur might be important for the creation of them. From this, we believe that boredom and aloneness can be considered as an important situation, especially for typically developing children. It seems that children create and interact with ICs when they want to be supported by or play with someone else. For example, it is believed that children create ICs when no one is around to play with (Taylor, 1999). Some children may play with their close-aged siblings at home, but only children or firstborns may not be able to do so. This is consistent with the finding that firstborn children were more likely to have ICs compared to second-born children or other succeeding children (for meta-analysis, see Moriguchi & Todo, 2018). Parents also think that playing with ICs may enable children to overcome of boredom and loneliness (Majors, 2013). Moreover, in India, where children spend less time alone, children were less likely to report ICs than US children (Klausen & Passman, 2007; Mills, 2003). Thus, if children spend lot of time alone and cannot access their peers, they might create and interact with their ICs to entertain themselves. However, there has been less empirical examination of situational influence where children cannot access their peers on the creation of ICs. Thus, our primary aim was to examine the impact of inaccessibility to peers on the prevalence of ICs.

The pandemic of COVID-19—the disease caused by a coronavirus (SARS-CoV-2)—has greatly influenced and changed our daily lives. In some countries, governments banned people from going outside for a certain period to prevent the virus from spreading. The Japanese government also declared a state of emergency on April 7, 2020 and publicly required people to stay home unless it was necessary. This continued until this measure was lifted on May 15, 2020 (except for urban areas such as Tokyo), hindering social connection not only in adults, but also in children. During this period, children were forcibly isolated from their peers, decreasing their opportunities to meet and play with them. For instance, the Japanese government asked schools for temporary closure from March 2, 2020. Preschools, nursery schools, and kindergartens were not included in this request, however, some of them decided to shut down temporarily. Some parents also voluntarily refrained from leaving their children at day-care centers. Therefore, children’s opportunities to meet and play with their peers decreased. If imaginary companions were stimulated by situational factors, some children might create and interact with ICs during COVID-19 pandemic. We hypothesized that more children will create and interact with ICs during COVID-19 pandemic compared to before the pandemic.

In summary, we aimed to examine the impact of situational factors by comparing the prevalence of ICs during and before the coronavirus (COVID-19) pandemic. We hypothesized that if decreased opportunities to meet and play with peers stimulate the creation and interaction with ICs (personified objects and invisible friends), the prevalence of ICs would be higher during the COVID-19 pandemic (April 2020) compared to before the pandemic (late September 2019).

## Methods

### Participants

We conducted online-based surveys (through Cross Marketing, Inc.) twice: the first investigation (Time 1) was conducted on September 28, 2019, and the second investigation (Time 2), on April 29, 2020. Thus, the latter was conducted during the time when the Japanese government publicly required people to avoid unnecessary outings. We did not plan the Time 2 survey when we conducted the Time 1 survey. As a result, it was highly possible that we could not track most participants in Time 2, and the sample size might have been unbalanced between the age groups. Children’s age and gender were important variables in the current study. Thus, we decided to collect participants cross-sectionally to obtain reliable data, so the participants who took part in Time 1 were not included in Time 2. Each survey investigated 560 Japanese primary caregivers who cared for children aged 2–9 years. There were 70 participants in each age group, and the gender ratio was even in all groups. The demographic information of the participants is shown in Table 1: children’s age in months, sex ratio (ratio of girls to boys), number of siblings, parents’ age, sex ratio (ratio of mothers to fathers), number of family members, parental level of education, and family income did not differ between the Time 1 and Time 2 samples.

**Table 1.** Demographic Information of the Sample.

Variables	Before the pandemic Mean (*SD*)	During the pandemic Mean (*SD*)	Statistics
Caregivers’ age (years)	38.67 (5.95)	39.06 (5.81)	*t* = −1.10, *p* = .273
The ratio of mothers	92.50%	89.46%	*χ*^2^ = 2.79, *p* = .095
The number of family member	3.95 (0.95)	3.95 (1.04)	*t* = 0.03, *p* = .976
Education level^a^	Junior high school: 10 High school: 124 Vocational school: 89 Technical college: 11 Junior college: 78 4-year-college: 223 Graduated school: 23 Others: 2	Junior high school: 9 High school: 129 Vocational school: 89 Technical college: 8 Junior college: 85 4-year-college: 214 Graduated school: 24 Others: 2	*χ*^2^ = 1.13, *p* = .992
Family income^a^	< 2,000,000 yen: 17 < 4,000,000 yen: 68 < 6,000,000 yen: 153 < 8,000,000 yen: 110 < 10,000,000 yen: 68 < 12,000,000 yen: 18 < 15,000,000 yen: 15 < 20,000,000 yen: 9 ≧ 20,000,000 yen: 5 Not Answered: 97	< 2,000,000 yen: 24 < 4,000,000 yen: 75 < 6,000,000 yen: 128 < 8,000,000 yen: 105 < 10,000,000 yen: 64 < 12,000,000 yen: 31 < 15,000,000 yen: 18 < 20,000,000 yen: 8 ≧ 20,000,000 yen: 4 Not Answered: 103	*χ*^2^ = 8.07, *p* = .527
Children’s age, (months)	71.41 (27.43)	71.95 (27.80)	*t* = −0.32, *p* = .746
The ratio of girls	50.00%	50.00%	*χ*^2^ = 0, *p* = 1
The number of, siblings	1.87 (0.78)	1.82 (0.84)	*t* = 1.18, *p* = .238

*Note*. a. Categorical variables. The number of participants were shown.

### Procedure

The questions were identical at Time 1 and Time 2. After providing the demographic information, each participant reported whether their children spontaneously had or have had POs and IFs. We first asked participants whether their children spontaneously used toys. If the children currently used toys spontaneously and daily, they were asked to read an episode describing POs. This question was included because the concept of ICs is not widespread in Japan (Moriguchi & Todo, 2019; Yamaguchi & Moriguchi, 2020). After reading the episode, participants were asked to judge whether their children’s toys were similar to the POs. If they said yes, they were also asked (1) the age of onset and offset (if applicable) using six-month interval scale (i.e., at the age of 6 months, at the age of 12 months, …, at the age of 114 months), (2) the intensity of the attachment (rated on a 10-point Likert scale), and (3) how the toys were used (i.e. suck, rub their skin, hold, speak to, behave as if listening to the object, attributing personality: rated on 5-point Likert scales) for each object.

Participants were also asked to read another episode on IFs. They then reported whether their children had IFs. If they answered yes, they were also asked (1) the age of onset and offset (if applicable), and (2) the intensity of their children’s attachment to them.

In the Time 2 survey, we added a question to check whether children’s opportunities to meet their peers decreased. Participants were required to answer how frequently their children attended preschool or school (days per week), 3 months before (January 2020), and at the time of the survey (during the pandemic). Although we could not compare them with the first investigation (September 2019), this helped check if social isolation occurred.

### Data Analysis

First, we conducted a Wilcoxon’s rank sum test to check whether children’s opportunities to meet their peers significantly decreased. We expected that children would go to preschools or schools in April 2020 less frequently than they did 3 months ago.

Next, we counted the number of children who currently had ICs as current reports would be more reliable than retrospective ones. We identified ICs based on the caregivers’ reports. Although we did not interview children directly, a meta-analysis showed that the reporter did not affect the prevalence of ICs (i.e., caregivers or children, Moriguchi & Todo, 2018).

To ensure that the characteristics of POs did not change during and before the COVID-19 pandemic, we compared the behaviors towards their POs between the two samples. We conducted Wilcoxon rank sum tests for each behavior (suck, rub their skin, hold, speak to, behave as if listening to the object, and attributing personality).

To compare the prevalence of ICs before COVID-19, we conducted logistic regression models for each object (i.e. POs and IFs). The response variable was the status of each object (e.g. currently had POs = 1, not currently had POs = 0). To control for the effects of variables that potentially influence the effect of the COVID-19 pandemic, we included children’s gender, age in months, number of siblings, number of family members, parental age, parental gender, parental education level, and family income as fixed effects, as well as the effect of the COVID-19 pandemic (before vs. during the pandemic). The variance inflation factors (VIFs) were lower than 2.04. We expected that the effect of the COVID-19 pandemic would significantly explain the variance in the status of ICs (POs and IFs).

To check whether the inaccessibility in playing with peers stimulated the creation of imaginary companions, we counted the number of children who created their ICs within last 6 months (we labelled them as “new creator”), and compared them between Time 1 and Time 2.

Finally, we also conducted logistic regression models for each object to explore important demographic variables that associate the creation of ICs (POs and IFs), by contrasting children who currently had ICs (= 1) and those who never had ICs (= 0). We selected the best predictive model by Akaike Information Criterion (AIC) to estimate the precise impact of each demographic information on the creation of ICs.

## Results

### Children’s Opportunities to Meet Their Peers

A Wilcoxon’s rank sum test showed that children went to their preschools or schools less frequently during the pandemic (*M* = 0.82 days per week, *SD* = 1.81) than 3 months before (*M* = 3.90 days per week, *SD* = 2.08), *W* = 257,563 *p* < .001, Cliff’s *d* = .64,395%CI [.594, .687]. This indicated that children’s opportunities to meet their peers or friends decreased during the pandemic compared to before the pandemic.

### Behaviors Towards Children’s Attachment Objects

Children attributed personality traits to their POs less frequently during the pandemic compared to before, *W* = 2108, *p* = .044, Cliff’s *d* = .21,095%CI [-.005, .407]. Other behaviors were comparable between the two samples, *W*s < 2043, *p*s > .061 (Table 2).

**Table 2.** Children’s Behaviors Toward Their Personified Objects.

Variables	Before the pandemic	During the pandemic	*W*	*p*	Cliff’s delta
suck	1.74 (1.20)	1.40 (0.74)	1935.5	.218	.111
rub their skin	3.44 (1.37)	3.64 (1.41)	1565	.339	−.101
hold	4.49 (0.83)	4.46 (0.95)	1752.5	.948	.006
speak to the object	4.58 (0.88)	4.54 (0.82)	1825	.585	.048
behave as if listening to the object	4.58 (0.96)	4.30 (1.10)	2043	.061	.173
attributing personality	4.02 (1.41)	3.70 (1.26)	2108	.044	.210

*NOTE*. Means, standard deviations (in the parentheses), and the results of Wilcoxon rank sum tests were shown.

### Prevalence of Imaginary Companions

**Figure 1.**
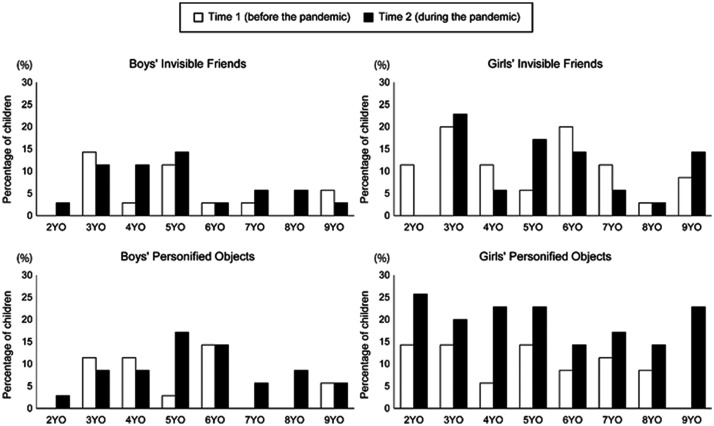
Prevalence of Imaginary Companions in Time 1 and Time 2 Survey.

The number of children with ICs in the Time 1 sample, and the Time 2 sample is shown in Table S1 of the Supporting Information. Children were strongly attached to most POs (*M* = 8.47, *SD* = 1.40). The logistic regression model showed that the COVID-19 pandemic affected the prevalence of POs, even when the other variables (except for parental education level because the model failed to estimate the parameter) were controlled, *G*^2^(1) = 12.00, *p* < .001, *R*^2^ = .013, 95%CI [.003, .029]; the effects of other variables are shown in Table 3. This indicated that we found more children with POs during the pandemic than before, *OR* = 2.01, *p* < .001, [1.34, 3.00]. The model that included all demographic variables (except for family income because the model failed to estimate the parameter) showed the prevalence of IFs was not affected by the pandemic of COVID-19, *G*^2^s (1) = 0.03, *p* = .853, *R*^2^ < .001 [< .001, .005] (Figure 1, see also Table 4).

**Table 3.** The Results of Logistic Regression Model for the Prevalence of Personified Objects.

Variables	Estimate	*SE*	*z*	*p*	*R* ^2^	*95%CI*	
						*Lower*	*Upper*
Children’s gender *G*^2^(1) = 16.29, *p* < .001 (reference = Boys)							
Girls	0.813	0.207	3.931	< .001	.030	.014	.053
Children’s age in month *G*^2^(1) = 4.83, *p* = .028							
	−0.009	0.004	−2.184	.029	.002	.000	.010
Number of siblings *G*^2^(1) = 3.54, *p* = .060							
	−0.349	0.185	−1.884	.060	.004	.000	.015
Parent’s age *G*^2^(1) = 6.14, *p* = .013							
	0.046	0.018	2.484	.013	.001	.000	.008
Parent’s gender *G*^2^(1) < 0.01, *p* = .980 (reference = Father)							
Mother	0.008	0.347	0.024	.980	.000	.000	.005
Number of family members *G*^2^(1) = 0.21, *p* = .650							
	0.064	0.140	0.460	.646	.001	.000	.007
Family income *G*^2^(9) = 13.81, *p* = .129 (reference = “< 2,000,000 yen”)							
<4,000,000 yen	−0.275	0.580	−0.474	.635	.000	.000	.005
<6,000,000 yen	−0.074	0.534	−0.139	.889	.001	.000	.007
<8,000,000 yen	−0.397	0.556	−0.715	.475	.000	.000	.005
<10,000,000 yen	0.244	0.557	0.438	.661	.001	.000	.008
<12,000,000 yen	0.038	0.648	0.059	.953	.000	.000	.005
<15,000,000 yen	0.798	0.674	1.184	.236	.002	.000	.011
<20,000,000 yen	−0.923	1.175	−0.785	.432	.000	.000	.006
>20,000,001 yen	1.814	0.908	1.998	.046	.004	.000	.015
Not Answered	0.112	0.545	0.205	.837	.001	.000	.008
The COVID-19 pandemic *G*^2^(1) = 12.00, *p* < .001 (reference = Before the pandemic)							
During the pandemic	0.696	0.205	3.395	< .001	.013	.003	.029

Note. AIC = 755.11. *P*-values are not adjusted for family income.

**Table 4.** The Results of Logistic Regression Model for the Prevalence of Invisible Friends.

Variables	Estimate	*SE*	*z*	*p*	*R* ^2^	95%CI	
						Lower	Upper
Children’s gender *G*^2^(1) = 0.19, *p* = .659 (reference = Boys)							
Girls	0.075	0.170	0.441	.659	.000	.000	.005
Children’s age in month *G*^2^(1) = 0.33, *p* = .568							
	0.002	0.003	0.571	.568	.000	.000	.006
The number of siblings *G*^2^(1) = 1.52, *p* = .217							
	−0.190	0.154	−1.237	.216	.001	.000	.009
Parent's age *G*^2^(1) = 2.37, *p* = .123							
	−0.025	0.017	−1.532	.126	.002	.000	.011
Parent’s gender *G*^2^(1) = 0.49, *p* = .483 (reference = Father)							
Mother	−0.219	0.307	−0.712	.476	.000	.000	.005
Parental level of education *G*^2^(7) = 5.98, *p* = .542 (reference = Junior high school)							
High school	−0.065	0.659	−0.099	.921	.000	.000	.005
Junior college	−0.114	0.675	−0.169	.866	.000	.000	.005
Vocational college	−0.515	0.681	−0.756	.450	.001	.000	.007
Technical college	0.307	0.853	0.359	.719	.000	.000	.005
4-year-college	−0.234	0.653	−0.358	.720	.000	.000	.005
Graduated school	0.370	0.730	0.507	.612	.000	.000	.006
Others	0.449	1.323	0.340	.734	.000	.000	.005
Number of family members *G*^2^(1) = 0.06, *p* = .805							
	0.029	0.117	0.249	.804	.000	.000	.005
The COVID-19 pandemic *G*^2^(1) = 0.03, *p* = .853 (reference = Before the pandemic)							
During the pandemic	0.031	0.170	0.185	.853	.000	.000	.005

*Note*. AIC = 962.19. *P*-values are not adjusted for parental level of education.

Although we found a larger number of children had POs significantly during the COVID-19 pandemic compared to before the pandemic, the number of new creators of POs were comparable between before (*n* = 19) and during the pandemic (*n* = 21, binomial test *p* = .875). The number of new creators of IFs were also comparable between before (*n* = 10) and during the pandemic (*n* = 5, binomial test *p* = .302).

### Important Demographic Information That Predicted the Creation of Imaginary Companions

We also analyzed the best models that were chosen based on Akaike Information Criterion (AIC) to extract important factors for the prevalence of ICs. For POs, the model with best fit included children’s age, children’s gender, number of siblings, parent’s age, and the pandemic as variables (AIC = 736.89). The prevalence of personified objects was significantly predicted by children’s age (*β* = -0.09, *SE* = 0.004, *p* = .030), children’s gender (*β* = 0.83, *SE* = 0.21, *p* < .001), number of siblings (*β* = -0.28, *SE* = 0.13, *p* = .032), parent’s age (*β* = 0.05, *SE* = 0.02, *p* = .006), and the pandemic (*β* = 0.71, *SE* = 0.20, *p* < .001, each effect was shown in Table S2 in Supporting Information). For IFs, the best model included children’s age, children’s gender, and parent’s gender as variables (AIC = 632.76). The prevalence of IFs was significantly predicted by children’s gender alone, *β* = 0.61, *SE* = 0.22, *p* = .006 (other effects were shown in Table S2 in Supporting Information).

## Discussion

To our best knowledge, this is the first study examining the impact of the inaccessibility to play with peers on the prevalence of children’s imaginary companions (ICs) with large sample size. Children’s opportunities to meet their peers or friends may decrease during the pandemic compared to before the pandemic. We found that larger number of children had personified objects (POs) during the COVID-19 pandemic compared to before the pandemic, even after accounting for the effect of other demographic information. However, the number of children who recently created their POs was comparable between the Time 1 sample and the Time 2 sample.

Our interpretation is that our participants’ children did not create ICs, neither POs nor invisible friends (IFs), in response to COVID-19 pandemic. In other words, we could not obtain strong evidence that inaccessibility to peers stimulates creation of ICs. However, it is still possible that children restarted play with their POs during the COVID-19 pandemic. Moriguchi and Todo (2019) reported that the percentage of Japanese children who currently had POs decreased with age: 16% at the age of five, 11% at the age of six, and only 7% at the age of seven. In our sample, the percentage of children who currently had POs was almost constant. Even among the caregivers of nine-year-olds, 14% reported that their children had POs during the pandemic. This suggested that children who already ceased to play with POs might restart the play during the pandemic. Given that elementary schools were shut down earlier (March 2, 2020) than preschools, school-aged children struggle from boredom or loneliness more than preschoolers or kindergarteners, and they might play with their POs to entertain themselves (Majors, 2013; Taylor, 1999).

Another interpretation is that children might play with their POs more frequently, and this increased opportunities for caregivers to notice the existence of POs. Some caregivers may have thought that their children ceased to play with their POs. Witnessing their child playing with their POs during the pandemic might have helped them realize that their children still had POs. This might explain why caregivers reported that their children attributed personality traits to personified objects less frequently during the pandemic compared to before the pandemic.

Increased opportunity to witness children’s play might also increase the likelihood to identify POs because caregivers must observe children’s play multiple times to identify the objects as POs (i.e., attributing consistent person-like qualities to their special objects for at least a month). If caregivers had less opportunity to observe their children’s play, they might consider their play just as mere pretend play, such as playing house.

Both interpretations suggested that children might play with their POs more frequently when they cannot play with their peers. Related to this suggestion, we found that number of siblings negatively predicted the prevalence of POs, suggesting that solitary situation was important to interact with their POs. Our interpretation is consistent with the idea that enough time to play alone might be important to interact with ICs (Klausen & Passman, 2007). Thus, the inaccessibility to play peers might stimulate interaction with POs.

Despite of our expectations, we did not detect an increase in the prevalence of IFs. IFs were more likely to be treated as horizontal relationships than POs (vertical relationship; the child cared for, or taught, or disciplined, or guided the companions) (Gleason et al., 2000). If children created ICs to cope with the deprivation of real peers, IFs might be more appropriate than POs. Although Moriguchi and Todo (2019) showed that about 27% of Japanese children’s POs were of the same age as them (about 52% were younger than the children), they did not assess child-ICs relationships directly. Moreover, recent studies did not support the relation that IFs were related to horizontal relationships and that POs were vertical relationships in a Chinese sample (Lin et al., 2018, 2020) and US sample (Gleason & Kalpidou, 2014). Thus, it is possible that POs might also function as children’s playmates. Future studies should interview children and assess the relationship between children and ICs in Japanese sample. Another possibility is that the required ability or condition to create IFs might be different from POs. A previous study showed that Japanese children were more likely to create POs than American and European children, although the prevalence of total ICs (POs plus IFs) was not different between cultures (Moriguchi & Todo, 2018). Unfortunately, the reason for this is still unclear. Moriguchi and Todo (2018) speculated that religion (e.g., Shintoism) was considered one of the possible factors. Japanese children might create POs because it is easier for them than the creation of IFs; future studies are needed to support this idea.

We should note that girls were more likely to have ICs (both POs and IFs) than boys, which is consistent with previous findings (e.g., Moriguchi & Todo, 2018). Figure 1 indicates that the difference in the prevalence of POs before and during the pandemic was especially evident among girls. This might be because of multiple factors. For example, research has demonstrated that girls are more likely than boys to choose stuffed animals or dolls for play (Todd et al., 2018). Additionally, girls are more likely than boys to cope with stress by seeking social support, although most of the research has involved children older than seven years of age, and the difference was greater in adolescents than children (for a review, see Rose & Rudolph, 2006). The latter might be related to our interpretation that girls were more likely than boys to interact with their POs during the COVID-19 pandemic. However, the reason why girls are more likely than boys to have ICs is not well understood. Therefore, clarifying the mechanism underlying this gender difference is necessary.

## Limitations and Conclusion

First, we adopted questionnaire survey and collected data online. Thus, there might be an active-participant bias. Moreover, this cross-sectional survey could not determine whether decreased opportunities to meet and play with real playmates caused creation of and interaction with ICs. Future studies should examine the situational factor through direct observation of children in the longitudinal study.

Second, although we proposed that the interaction with POs can be stimulated by both the children’s characteristics and the situational factors, we did not collect children’s characteristics in the current study. For example, given that IC can be regarded as a stress coping strategy (e.g., Taylor, 1999), children’s stress-related variables might be related to the children’s IC status. Further surveys are needed on this issue.

Third, related to the second point, environmental factors should have been more closely investigated. There might be several pathways to create and interact with ICs. For instance, children might create and interact with their ICs in response to stress. If so, parent-child conflict or parent-child relationships might be related to the prevalence of ICs. Recent reports suggest that stressors related to the COVID-19 pandemic, like job loss, financial strain, and food insecurity, influenced parent’s depression (e.g., Lawson et al., 2020; Roos et al., 2021). This led to psychological and physical maltreatment (Feinberg et al., 2021) and parent-child conflict (Russell et al., 2020). In contrast, some children might have interacted with their peers through online communication technologies like video chat, text messages, or social networking services during the pandemic. This possibly mitigated the impact of the pandemic on communication frequency with their peers, and consequently, the child might not need to play with their ICs. Future studies should control these factors to understand the association between inaccessibility to play with peers and children’s IC creation and interaction.

In conclusion, we showed that the prevalence of POs was higher during the pandemic of COVID-19 than before the pandemic. This finding is consistent with parents’ and researchers’ beliefs that children interact with ICs when the real playmate is not available (Taylor, 1999), and the playing with ICs might enable children to overcome times of boredom and loneliness (Majors, 2013).

## Supplemental Material

sj-pdf-1-ica-10.1177_02762366211034402 - Supplemental material for Did Children Interact With Their Personified Objects During the COVID-19 Pandemic? Click here for additional data file. Supplemental material, sj-pdf-1-ica-10.1177_02762366211034402 for Did Children Interact With Their Personified Objects During the COVID-19 Pandemic? by Masanori Yamaguchi and Yusuke Moriguchi in Imagination, Cognition and Personality
