# Supplementary material for: Did Children Interact With Their Personified Objects During the COVID-19 Pandemic?
Source: Imagin Cogn Pers. 2022 Mar;41(3):354–67. doi: 10.1177/02762366211034402 (PMC8350448; doi:10.1177/02762366211034402)
Supplement: sj-pdf-1-ica-10.1177_02762366211034402 - Supplemental material for Did Children Interact With Their Personified Objects During the COVID-19 Pandemic? [file sj-pdf-1-ica-10.1177_02762366211034402.pdf]

Did children interact with their personified objects during the COVID-19?

Supplemental Materials

Table S1. *The number of children who currently had imaginary companions.*

		Before the pandemic					During the pandemic				
Age	Gender	Current IFs	Current POs	Past IFs	Past POs	Neither	Current IFs	Current POs	Past IFs	Past POs	Neither
2-year-olds	Boys	0	0	2	1	32	1	1	0	1	32
	Girls	4	5	1	0	28	0	9	3	0	23
3-year-olds	Boys	5	4	3	2	24	4	3	4	1	25
	Girls	7	5	0	0	26	8	7	0	2	21
4-year-olds	Boys	1	4	3	1	26	4	3	2	1	27
	Girls	4	2	2	3	27	2	8	1	1	24
5-year-olds	Boys	4	1	1	1	28	5	6	3	3	24
	Girls	2	5	0	1	28	6	8	3	3	19
6-year-olds	Boys	1	5	6	0	24	1	5	5	3	24
	Girls	7	3	3	2	24	5	5	1	0	26
7-year-olds	Boys	1	0	5	2	29	2	2	3	0	29
	Girls	4	4	3	1	25	2	6	1	1	25
8-year-olds	Boys	0	0	1	0	34	2	3	4	1	26
	Girls	1	3	2	3	28	1	5	1	0	29
9-year-olds	Boys	2	2	2	5	27	1	2	2	1	30
	Girls	3	0	2	2	28	5	8	3	3	20

Note. POs indicates personified objects, and IFs means invisible friends.

Table S2. *The results of the best predictive logistic regression models for the prevalence of personified objects and invisible friends.*

variables	Estimate	*SE*	*z*	*p*	R^2	*95%CI*	
						Lower	*Upper*
**Personified objects (AIC = 736.89)**							
**Children's gender $G^2(1) = 17.48, p < .001$ (reference = Boys)**							
Girls	0.834	0.205	4.066	<.001	.011	.002	.027
**Children's age in month $G^2(1) = 4.76, p = .029$**							
	-0.009	0.004	-2.169	.030	.008	.001	.021
**Number of siblings $G^2(1) = 4.89, p = .027$**							
	-0.282	0.131	-2.151	.032	.003	.000	.013
**Parent's age $G^2(1) = 7.43, p = .006$**							
	0.048	0.017	2.756	.006	.002	.000	.012
**The COVID-19 pandemic $G^2(1) = 12.62, p < .001$ (reference = Before the pandemic)**							
During the pandemic	0.706	0.203	3.478	< .001	.002	.000	.010
**Invisible friends (AIC = 632.76)**							
**Children's gender $G^2(1) = 7.73, p = .005$ (reference = Boys)**							
Girls	0.612	0.224	2.728	.006	.007	.000	.020
**Children's age in month $G^2(1) = 2.65, p = .103$**							
	-0.006	0.004	-1.621	.105	.003	.000	.013
**Parent’s gender $G^2(1) = 2.85, p = .091$ (reference = Father)**							
Mother	-0.576	0.324	-1.777	.076	.001	.000	.007
